# Considerations for Cannabinoids in Perioperative Care by Anesthesiologists

**DOI:** 10.3390/jcm11030558

**Published:** 2022-01-22

**Authors:** Krzysztof Laudanski, Justin Wain

**Affiliations:** 1 Department of Anesthesiology and Critical Care, University of Pennsylvania, Philadelphia, PA 19104, USA; 2 School of Osteopathic Medicine, Campbell University, Buies Creek, NC 27506, USA

**Keywords:** cannabinoids, anesthesia, cannabidiol, tetrahydrocannabinol, neocannabinoids, perioperative management, review, human

## Abstract

Increased usage of recreational and medically indicated cannabinoid compounds has been an undeniable reality for anesthesiologists in recent years. These compounds’ complicated pharmacology, composition, and biological effects result in challenging issues for anesthesiologists during different phases of perioperative care. Here, we review the existing formulation of cannabinoids and their biological activity to put them into the context of the anesthesia plan execution. Perioperative considerations should include a way to gauge the patient’s intake of cannabinoids, the ability to gain consent properly, and vigilance to the increased risk of pulmonary and airway problems. Intraoperative management in individuals with cannabinoid use is complicated by the effects cannabinoids have on general anesthetics and depth of anesthesia monitoring while simultaneously increasing the potential occurrence of intraoperative hemodynamic instability. Postoperative planning should involve higher vigilance to the risk of postoperative strokes and acute coronary syndromes. However, most of the data are not up to date, rending definite conclusions on the importance of perioperative cannabinoid intake on anesthesia management difficult.

## 1. Introduction

Cannabinoids are defined as chemicals found in the *Cannabis sativa* plant, with tetrahydrocannabinol (THC) and cannabidiol (CBD) being the most mentioned compounds [1,2]. There are over 60 other naturally occurring cannabinoids [1,2,3,4,5,6]. Other plants such as Echinacea purpurea, Echinacea angustifolia, Acmella oleracea, Helichrysum umbraculigerum, Radula marginate and Kava contain lipophilic alkyl amides, which are considered cannabinoids based on their chemical structure resemblance and their ability to interact with cannabinoid receptors [7,8]. Finally, synthetic (neocannabinoids) and endogenous cannabinoid compounds are also known [9,10].

Structurally, cannabinoids resemble lipids, as all cannabinoids belong to the terpenes class [2,3,4]. These compounds are derivatives of the 5-carbon compound isoprene and constitute over 30,000 chemicals with common formula (C_5_H_8_)_n_ [2]. Cannabinoids and terpenes are responsible for a plant’s smell, ultraviolet protection, color, and structural support [4]. However, they are of increased interest because they possess several unique pharmacological properties [1,2,4,11,12,13,14,15]. These pharmacological potentials have been utilized in several ways, with recreational use comprising the majority of cannabinoid consumption [2,4,12,16,17]. FDA-approved indications for cannabinoid treatment include seizures, nausea, cancer pain, and certain neurological conditions [18,19,20]. Off-label medical treatments utilizing cannabinoids include treatments for headaches, schizophrenia, chronic pain, dementia, post-traumatic stress disorders, and many other conditions [21,22,23,24]. The increased presence of cannabinoids is of obvious concern for anesthesiologists, as more patients will likely consume them in increasing doses, potentially affecting anesthesia planning and execution.

This rising abundance of cannabinoid usage in the general public is somewhat juxtaposed by difficult access to reliable, evidence-based, up-to-date information about their clinical actions and properties [25]. One should appreciate a yin and yang approach to cannabinoids, considering the many different aspects of their narrative. Cannabinoids often exhibit opposite actions depending on the exact compound studied, their enantiomers, species used to study the compound, or overall health status [26,27,28,29,30]. Their social acceptance and legal approval vary greatly [25,31,32]. The scientific opinions about the use and danger of opioids often juxtapose one another, while the robust clinical evidence is frequently dated to the time before the “War on Drugs” was initiated in the 1980s [12,14,25,26,33,34]. There are also a significant number of claims in the media that are difficult to validate but influence patients’ consumption of cannabinoid products [17,19,25,35,36,37,38]. Patients often consume cannabinoids regardless of their efficacy [39,40]. Other confounding variables affecting the assessment of cannabinoid properties are the lack of compound standardization and the increasing variability in their strength and composition [17,19,35,36]. Frequently, natural cannabinoids are laced with other illicit substances causing modifications of their effects with significant clinical consequences [17,41,42].

The primary use of natural cannabinoids is centered around recreational and illicit use [2,17]. Neocannabinoids are frequently added as adulterators or potentiators to natural cannabinoid preparations [9,10,17,43]. Illicit or recreational marijuana use often affects individuals from disadvantaged backgrounds, thus adding to their illness burden while interacting with their primary treatment [24,38,44,45,46]. Finally, cannabinoid-based compounds are becoming more commonly approved and regulated in drugs such as Nabilone (Cesamet™), Cannabidiol (Epidiolex™), Nabiximols (Sativex™), Dronabinol (Marinol™, Adversa™, Syndros™, and Reduvo™), and Rimonabant/SR141716 (Acomplia™ and Zimulti™) [47,48,49,50].

Considering the complexity of the information about these compounds, an average anesthesia provider may find him/herself in need of a comprehensive synthesis of information pertaining to the execution of an anesthesia plan. Unfortunately, though prior reviews exist, they are often short in scope, noncomprehensive, frequently citing somewhat limited literature, focused on a specific population, or addressing a specific audience [51,52,53].

For the scope of this paper, we will focus on the possible effects of cannabinoids on the formulation of perioperative care in the adult population. For simplicity, we decided to use cannabinoids as the word covering all types of marijuana derivatives and similar synthetic structures unless specified otherwise in the text. This review aims to provide the reader with a comprehensive review of the potential ways cannabinoids and their derivatives may affect anesthesia planning and execution.

## 2. Types of Cannabinoids

**Figure 1:**
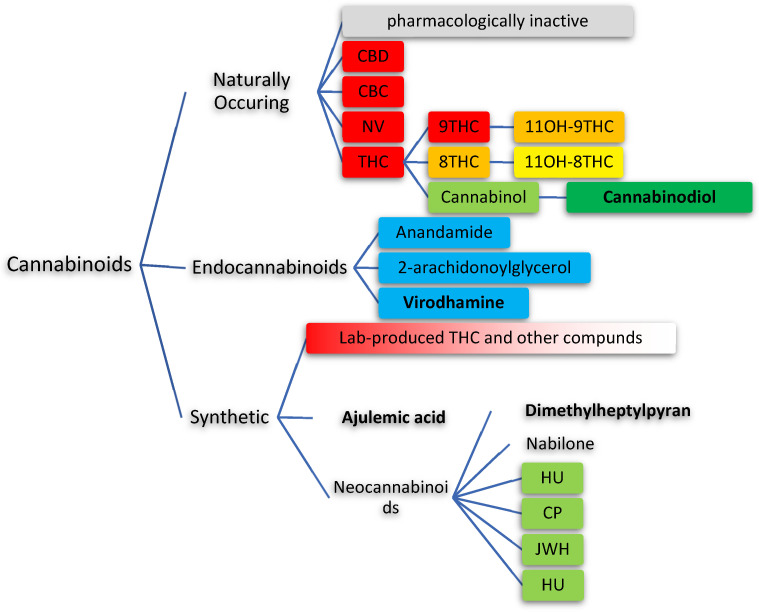
Division of cannabinoids depending on the source with color-coded psychoactive potency (red: most present, yellow: less present, green: not present; blue: endocannabinoids).

Cannabinoids can be grouped as synthetic, endogenous, or natural [2,4,54] (Figure 1). Natural cannabinoids are referenced to tetrahydrocannabinol (THC) as the standard, though multiple isomers having the same chemical formula exist (C_21_H_30_O_2_) [1,3,29]. The standard that all cannabinoids are referenced to is (−)-trans-Δ9-tetrahydrocannabinol (9-THC), the most potent psychoactive isomer [4]. On the other hand, (−)-trans-Δ8-tetrahydrocannabinol (8-THC) and hydroxylation products of both 8-THC and 9-THC, demonstrated decreasing psychoactive potency [3,55,56,57,58]. The focus on the psychoactive action of 9-THC led to the labeling of other cannabinoids as inert if they did not exhibit psychoactive activity. However, this is a misleading term, as psychoactive properties are not the only desirable trait, even if they are the most popular trait of these compounds. Several inert cannabinoids have not been characterized in depth despite interesting pharmacology [4,6,9,11,54,59]. To add to the complexity of the presented landscape, cannabinoids can be synthesized de novo [10,43]. These cannabinoids produced in the lab are called neocannabinoids [9,10,42,43]. To distinguish them from lab-originating THC, the term neocannabinoids is used [9].

## 3. Utilization of Cannabinoids

According to the National Survey on Drug Use and Health conducted in the USA in 2019, 48.2 million people ages 12 and up were marijuana users. 3.3 million were between ages 12 and 17, 12 million were between ages 18 and 25, and 33 million were aged 26 or older, according to the official tally [60]. Marijuana remains the most common illicit substance in the USA. Marijuana consumption increased by 6.5% from 2002 to 2019, especially in states that legalized the use of marijuana [16,17]. Neocannabinoids, synthetic analogs of naturally occurring cannabinoids, are used by individuals between 20 and 40 years old, especially in cases of fatal overdose [42,43].

These numbers are probably underestimated, as consumption of cannabinoids is frequently penalized and only recently significantly increased, following decriminalization in law and social acceptance [61]. Social acceptance varies by country and by state within the USA based on the rates of use within the population [33]. In the USA, recreational cannabinoids have become legalized in 18 states since 2012. This increased consumption drove the increased incidence of side effects [3,62,63]. Worldwide, several countries no longer penalize marijuana or cannabinoid consumption while others maintain cannabis compounds as illegal compounds [58]. In addition, higher concentrations of cannabinoid psychoactive compounds further complicate recreational and illicit use compared to the prior century [18,19,20]. As mentioned earlier, legal and societal background determines the likelihood that anesthesiologists will encounter individuals consuming cannabinoids for various reasons [12,28,37,38,64]. However, the increased prevalence of cannabinoid compounds is such that accidental intake has been reported in all ages, including newborns [65,66,67,68]. Intake of cannabinoids from FDA-approved medication remains a minuscule proportion of cannabis intake by the general population [47,48,49,50].

## 4. Source of Cannabinoids

Cannabinoids are natural compounds extracted from the Cannabis sativa plant, with flowers, buds, leaves, and stems being utilized to produce active compounds. Depending on the concentration of 9-THC, the plant is classified as hemp (below 0.3%), while a higher concentration is labeled as marijuana. This is an important distinction, as hemp is legal in the USA while marijuana depends on local state and federal regulation [33].

Several formulations of cannabinoids exist. Dry leaves are one way to consume, but the more refined products, inhaled psychoactive compounds, come from Cannabis sativa. These products are divided into solvent and solvent-free formulations. Solventless concentrates include kief, hash, rosin, bubble hash, distillates, and isolates with increasing 9-THC content. Hashish is a resin from the Cannabis sativa plant with a higher concentration of psychoactive compounds (3–52.9%) [31,35,36]. The solvent-based formulation uses a hydrophobic solvent to extract 9-THC and similar compounds. Hash oil is an oleoresin obtained from cannabinoids or hashish and contains up to ~45% of 9-THC [31,35,36]. CBD oil is divided according to 9-THC concentration [69]. Some claim that hemp oil should not contain cannabinoids because it is produced for mass consumption, yet testing for 9-THC is not routinely performed [30].

The preferable route for cannabinoid intake is oral, followed by inhalation, with intravenous intake being the least frequently used [1,3,70,71,72,73].

It is also increasingly likely that FDA-approved compounds with derivatives of cannabinoids will become more prevalent in the general population, especially in individuals with complicated and complex diseases, as most of these medications are not the first line of treatment [22,48,55,74]. They include Cesamet™, Epidiolex™, Sativex™, Marinol™, Adversa™, Syndros™, Reduvo™, Acomplia™, and Zimulti™ [47,48,49,50].

## 5. Natural Synthesis of Cannabinoids

Several routes exist for natural cannabinoids to be synthesized and metabolized (Table 1). In general, they undergo a synthesis from a precursor using specific synthetases, followed by hydroxylation, carboxylation, and glucuronidation [75,76].

Cannabigerolic acid (CBGA), an acidic form of cannabigerol (CBG), is the precursor of (−)-trans-Δ^9^-tetrahydrocannabinol, or cannabidiol (CBD) [14,19]. Consequently, there is a reverse relationship between the concentrations of 9-THC and CBD in natural compounds [19]. This opposition between 9-THC and CBD is one example of a yin and yang effect in the cannabinoid world, as they seem to have opposite effects [19,77,78]. CBD has no psychoactive effect and interferes with 9-THC at the cannabinoid receptor and metabolism level [79]. However, due to the pressures from illegal manufacturers and criminal activities, there is an increase in the active compound of 9-THC and a decrease in CBD, resulting in increased potency of preparations [19,20]. THC also undergoes a natural transformation into cannabinol (CBN) devoid of psychoactive potency [80]. CBD can be cyclized into 9-THC and 8-THC during pyrolysis [58,81,82,83]. In addition, one must keep in mind that pyretic transformation of cannabinoids yields several hundred compounds, similar to smoking nicotine-based products or THC [82].

Under the nominal condition, 9-THC is converted into 11-hydroxy-Δ^9^THC (11-OH-THC), which is less potent than 9-THC [76]. However, if 9-THC is taken orally, more 11-OH-THC may be delivered after the transformation of 9-THC into this metabolite [76]. In addition, 9-THC can also break down into CBN, a nonpsychoactive metabolite [84].

Cannabichromene (CBC) is a naturally occurring, nonactive phytocannabinoid with unknown endogenous activity [85]. Cannabicitran (CBT) and cannabidivarin (CBDV) are phytocannabinoids with inconsistently described receptor affinity, metabolism, and natural biological activity [86,87,88].

## 6. FDA-Approved Cannabinoid Formulation

Four cannabinoid medications were approved for use in the USA by the FDA, but one regulatory permit has been withdrawn [110] (Table 1).

Dronabinol (Marinol™, Adversa™, Syndros™, and Reduvo™) is an FDA-approved drug that is a synthetic THC prepared in 2.5 mg, 5 mg, or 10 mg. Dronabinol is effective as a partial agonist of cannabinoid receptors CB1 and CB2. It is used as an appetite stimulant and was tested as a treatment for AIDS- and cancer-related anorexia [103,104]. After a six-week study, patients using the drug showed significant improvement in appetite and the drug is now widely used for anorexia/weight gain disorders [103,104,107]. Another study was held to compare the effectiveness of cannabis extract (CE) instead of delta-9-tetrahydrocannabinol (9-THC) as opposed to a placebo. In this study, 164 patients were treated with 1 mg of cannabidiol, 2.5 mg of THC, or a placebo by mouth for six weeks. An independent review board concluded that there was an insufficient difference between compounds [105]. Some suggest that the drug may be effective in obstructive sleep apnea, but no definite follow-up study was conducted after the initial report [106,107]. Marinol was tested for drug interactions with cytotoxic agents, anti-infective agents, sedatives, and opioid analgesics without showing any significant interactive events.

Cannabidiol (Epidiolex™) has concentrated cannabidiol (100 mg/mL) and is used to treat seizures in infantile patients with Dravet Syndrome and Lennox–Gastaut syndrome [49,89]. CYP3A4 and CYP2C19 metabolize Epidiolex^TM^, but the specific mechanism of action which makes cannabidiol an anticonvulsive agent is unknown [49]. Because of this, coadministration with strong CYP3A4 or CYP2C19 inhibitors will increase cannabidiol plasma concentrations, while coadministration with strong CYP3A4 and CYP2C19 inducers will decrease cannabidiol plasma concentrations, both leading to possible adverse effects or drug inefficiency. Like most antiepileptic medications, cannabidiol should not be stopped abruptly. In addition, some reports suggest an increased incidence of pulmonary infection secondary to CB2 interaction and immunosuppression [90,91,92].

Nabiximols (Sativex™) is a combination drug delivering a dose of 2.7 mg THC and 2.5 mg CBD with each spray and is approved to treat dry mucosa in patients with multiple sclerosis [102,111]. Some have suggested using the drug to manage chronic pain, but the medication did not meet this goal [112]. Nabiximols (Sativex™) has a good safety profile, with drowsiness and dizziness being the most common side effects.

Nabilone (Cesamet™) is a synthetic cannabinoid mimicking THC. It has been approved to treat neuropathic and other types of pain, but its impact is relatively small [99,100,113]. Consequently, nabilone is utilized as a pain adjunct. A clinical trial demonstrated its effectiveness against chemotherapy-related nausea in certain regiments [97,101,114]. The expanding indications of nabilone may include Alzheimer’s disease, Parkinson’s disease, and inflammatory bowel disease in the future [92,95,115]. Nabilone can stimulate appetite during treatment with 0.5 mg nabilone/2 weeks followed by 1.0 mg nabilone/6 weeks, with a subsequent increase in caloric intake of 342 kcal and an increased carbohydrate intake of 64 g [101]. The most common side effects mimic THC’s and include euphoria and dizziness [100,101]. No comparison to dronabinol effectiveness in terms of appetite stimulation is available.

Rimonabant/SR141716 (Acomplia™ and Zimulti™) is the only CB1 receptor antagonist. It was briefly approved as an antiobesity medication, but it was withdrawn due to serious side effects, including psychosis. Currently, this active compound has only experimental application [108].

## 7. Mechanism of Action

Several receptors for cannabinoids exist (Table 2) [116].

The two main cannabinoid receptors are cannabinoid receptor 1 (CB1) and cannabinoid receptor 2 (CB2), both of which are G-protein-coupled receptors (GPCR) on the cell surface [117,149].

The human CB1 receptor is encoded by the *CNR1* gene and consists of 472 amino acids [124]. The CB1 receptor is also found in rats and mice and consists of 473 amino acids with 97–99% amino acid sequence identity to humans, suggesting high structural homology. CB1 receptors are most abundant in the central nervous system, specifically in the cortex, hippocampus, basal ganglia, and cerebellum, contributing to cannabinoids’ effects on memory, cognition, movement, and nociception [150,151]. In addition, CB1 receptors are found in the intestine, liver, pancreas, immune system, heart, vascular system, and reproductive system but at lower receptor density [151]. CB1 receptors can also form homo- and heterodimers with other CB1 receptors and GPCRs to modulate receptor signaling [124]. Various neural functions have been suppressed in CB1 knock-out mice or the presence of a CB1 antagonist (SR141716A). Specifically, blocking the CB1 endocannabinoid receptor can suppress appetite and feeding behaviors [150], the extinction of aversive memories, cerebellum-dependent discrete motor learning, drug addiction, and neuroprotection following closed head injuries [123,150,152,153].

The human CB2 receptor is encoded by the *CNR2* gene and consists of 360 amino acids, sharing only 44% sequence homology to the CB1 receptor at the protein level. The CB2 receptor also shows greater variation amongst different species when compared to the CB1 receptor. The amino acid sequence homology of the CB2 receptor among humans and rodents is just above 80% [154]. CB2 receptors are most abundant on immune cells, specifically B-cells and natural killer (NK) cells, where stimulation of these receptors can modulate immune function [155]. Less pronounced receptor densities of CB2 are found in the adrenal gland, lung, myocardium, vascular smooth muscle, testis, prostate, bone, and some tumors [151,155]. CB2 receptors are particularly important for immune system function, specifically B-cells and natural killer (NK) cells [150]. They also play a role in seizure threshold and behavioral regulation [156].

THC serves as a partial agonist for CB1 and CB2. Endocannabinoids, which are lipid chemicals synthesized by humans and animals, serve as agonists and antagonists for the CB1 and CB2 receptors [150,151,154,157]. Common endocannabinoids that function as agonists are arachidonoylethanolamide (AEA) and 2-arachidonoylglycerol (2-AG), while common antagonists are sphingosine and desmopressin. When THC binds, a conformational change occurs in the transmembrane receptor, causing an interaction with the G-protein, allowing the release of guanosine diphosphate (GDP) from the G αi-sub-unit followed by the binding of guanosine triphosphate (GTP). The binding of GTP causes the activation of the G-protein, allowing the GTP-ai subunit to dissociate from the beta-gamma unit. The GTP-ai subunit inhibits adenylate cyclase, resulting in a decrease in cyclic adenosine monophosphate (cAMP) and subsequently lowering intracellular Ca^2+^ concentrations [79]. Compared to THC, CBD also acts as a partial agonist, but its binding to CB1 and CB2 is relatively weak. There is also significant dimerization between CB1 and CB2, resulting in different activation patterns depending on the agonist type, resulting in differential activation of G-couple protein, MAPK, and β-arrestin [158].

Studies have shown that CBD can bind to the serotoninergic 5-HT_1A_ receptor (5-HT_1A_) and opioid receptors, specifically the μ-opioid receptor (MOR) and δ-opioid receptor (DOR), which all function similarly to the cannabinoid receptors in their inhibition of adenylate cyclase [157]. The interaction with 5-hydroxytryptamine (5-HT_1A_) is another potential interaction with cannabis compounds. These interactions with the receptor in basal ganglia may be responsible for a potentially beneficial effect on motor function in some neurodegenerative motor disorders [13,14,159]. Similar interaction in striate may result in the antinausea and antivomiting effect of the cannabinoids [55,133,160]. The agonistic effect of 5-HT_1A_ located on the ventral medial prefrontal cortex may modulate the antidepressant effect of cannabinoids, but CB1 is often required to work synergistically to attain antianxiolytic antidepressant effects [132,161,162].

The TRPV receptor, capsaicin, and the vanilloid receptor are encoded by the TRPV gene and are involved in temperature regulation and pain sensation [134,135,136,137,138]. They have also been linked to decreasing the ability to make new memories by depressing long-term potentiation (LTP). Capsaicin is an agonist for this receptor used in most experimental studies [163]. Cannabinoid ligands acting via CB1 and TRPV1 can suppress inflammation and are particularly important for the performance of the endogenous cannabinoid system [120,141].

Some studies demonstrate cannabinoids interacting with alpha two receptors [14]. This interaction is linked to vasodilatory and antianxiolytic effects [127,128,129]. In addition, experimental data suggest the involvement of this receptor in cannabinoids’ anticonvulsant activity [130].

One should also be aware that other components of cannabinoids may exercise metabolic activity. For example, β-Myrcene is the most found terpene in modern Cannabis chemovars in the USA and is linked to sedation after intake [54,164,165]. In addition, α-pinen inhibits acetylcholinesterase and may be responsible for intoxication [166,167].

## 8. Pharmacodynamics and Pharmacokinetics

The absorption of cannabinoids can vary based on the route of administration. A rapid increase in the serum level of cannabinoids and the penetration into the brain are key components of the drug’s abusive potential.

Administering through inhalation or vaporization allows for rapid delivery into the bloodstream, causing THC and CBD plasma concentrations to peak within 3–10 min [76,168]. Following inhalation, the bioavailability of THC ranges from 10 to 85%, while the bioavailability of CBD averages 31%, both of which are dependent on inter- and intrasubject inhalation characteristics (number of puffs, the smoke volume, and amount of time smoke is held inside the lungs) [76,169]. Furthermore, pyrolysis may occur during smoking and vaping, resulting in CBD to THC conversion [81,83]. Smoking results in inhalation of combustion products similar to the mechanisms seen during tobacco consumption, but the significance of this is unclear [170,171]. Theoretically, vaping results in a rapid increase in THC without additional side effects related to smoking [172]. However, vaping risk factors were recently appreciated [29,173]. Injecting cannabinoids generated the fastest and highest peak, but it is uncommon [73,76]. Injecting cannabinoids may result in endocarditis and other infectious diseases, but this is highly unlikely, as the intravenous route is negligible for these compounds. THC and CBD have a bioavailability of roughly 6% when orally administered due to their lipophilic structures, variable gut absorption, and extensive hepatic first-pass metabolism, with plasma concentrations within the pharmacodynamics therapeutic range for 2 to 6 h [76,79,168,174,175]. The sublingual formulation of THC and CBD (Sativex^TM^) allows rapid absorptions and avoids hepatic first-pass metabolism, producing plasma levels higher than oral administration but less than inhalation administration [176]. Transcutaneous administration serves as another route to bypass hepatic first-pass metabolism and follows zero-order kinetics, but THC and CBD absorption decrease due to their hydrophobic structures and resistance from the subcutaneous skin layer [177,178]. Studies show that CBD is 10x more permeable in transcutaneous administration when compared to THC, indicating CBD has a more polar structure than THC [10,77,168,174,177].

Once in the bloodstream, 90% of THC and CBD are distributed to the plasma, while the remaining 10% are distributed to red blood cells [175]. Both THC and CBD have a distribution volume (*V*_d_) of 3.4 l kg^−1^ (calculated following inhaled administration) and *V*_d_ of ~32 l kg^−1^ (calculated following intravenous administration, despite the fact 95–99% is protein bound to lipoproteins in the plasma). THC and CBD are initially taken up by highly vascularized tissues such as the lungs, heart, brain, liver, mammary gland, fetus, adrenal cortex, and pituitary gland. When administered intravenously, only around 1% of THC is found in the brain at peak psychoactivity, which could be caused by the high perfusion rate within the brain moving THC in and out. The THC metabolite 11-hydroxy- Δ9tetrahydrocannabinol (11-OH-THC) is found in higher quantities in the brain when compared to the unmetabolized THC compound, suggesting the role 11-OH-THC may have in the effects experienced with THC [77,78,179]. The increased uptake of 11-OH-THC in the brain may be due to its lower plasma protein binding or the hydroxylated metabolite’s ability to pass through the blood brain barrier [76]. With chronic exposure to THC and CBD, eventually the chemicals make their way into adipose tissue, which serves as the long-term storage site.

The liver primarily metabolizes THC via cytochrome P450 (CYP-450) isozymes CYP2C19 and CYP3A4. Some THC can be metabolized outside the liver in tissues that express CYP-450, such as the brain, but the liver serves as the main site of metabolism via its microsomal system [3,168]. Isozymes CYP 450 2C9, 2C19, and 3A4 are involved in the phase-in oxidation of THC, generating hundreds of different metabolites [10,76,77]. Specifically, hydroxylation at the C9 carbon of THC generates 11-OH-THC, the most prominent product following the first round of oxidation. 11-OH-THC serves as an equipotent metabolite and a contributor to psychoactive properties of THC, with blood plasma levels of 11-OH-THC peaking roughly 13 min after smoking [76]. Subsequent oxidation of 11-OH-THC forms the inactivated product THC-COOH which undergoes Phase II metabolism, mainly consisting of glucuronidation into metabolites ready for excretion [76,174]. About 65% of THC metabolites are excreted within the feces, while the remaining 25% of metabolites are excreted in the urine [76,174]. Overall, the terminal half-life of THC is estimated to be between 25 and 36 h [179].

CBD is metabolized by CYP2C19, CYP3A4, CYP1A1, CYP1A2, and CYP2D6 [174]. Like THC, CBD C7 carbon is hydroxylated by isoenzymes CYP2C19 and CYP3A4, generating 7-OH-CBD [76]. The liver further breaks down 7-OH-CBD through glucuronidation, forming metabolites excreted in the feces and urine [76]. However, unlike THC, some CBD is able to be excreted in the feces unchanged [76,174]. As a result, the overall terminal half-life of CBD is shorter than that of THC, ranging from 18 to 32 h [174]. A relatively longer elimination half-life is observed in heavy users of THC and CBD, which is attributed to slow redistribution from deep storage compartments such as within adipose tissues [168].

The metabolism of cannabinoids has several important implications. First, there is extensive interaction with several drug classes on metabolism. Cannabinoid metabolism is usually prolonged in terms of metabolite removal. Since these metabolites are used to test for cannabinoid exposure, such as in a urine toxicology screen, it remains difficult to distinguish acute versus chronic versus accidental exposures [180]. There is a way to assess the level of 9-THC in the blood, but it is laborious and time-consuming.

### Tolerance Development

Chronic administration of cannabinoids over time reduces CB1 receptor density and coupling efficiency, resulting in tolerance to the acute effects of THC such as memory disruption, locomotion impairment, and its analgesic effect [88]. The first stage of desensitization of the CB1 receptors is uncoupling the G-protein receptor to the G αi-sub-unit, resulting in decreased receptor activation via β-arrestin involvement [121,181,182,183]. In rat studies, chronic administration of THC resulted in significant desensitization of CB1 receptors in different brain regions. While every brain region showed some desensitization to chronic administration of THC, different areas within the brain showed higher levels of desensitization as well as time required for desensitization [181,182]. The area of the brain showing the highest degree of desensitization (75%) and the fastest rate of desensitization (3 days) was the hippocampus [181]. Regions of the brain showing slower rates of desensitization were the cerebellum (7 days) and the globus pallidus (14 days) [181,184]. With different brain regions having variations in rate and degree of desensitization, tolerance to the behavioral effects of THC is developed over different periods. The idea of tolerance toward cannabinoid use can be viewed as beneficial because tolerance can serve as a protective response toward unwanted cannabinoid effects [88].

## 9. Endogenous Cannabinoid System

The identification of CB1 and CB2 receptors later led to the discovery of the endocannabinoids anandamide (AEA) and 2-arachidonoylglycerol (2-AG). Both AEA and 2-AG can be synthesized on demand from arachidonic acid within cellular membranes [185]. AEA and 2-AG function as endogenous ligands for the CB1 and CB2 receptors, producing effects similar to exogenous THC. 2-AG has full agonist activity toward CB1 and CB2 receptors with low to moderate affinity, while AEA has partial agonist activity toward CB1 but not CB2 receptors with high affinity [154]. Since AEA and 2-AG both consist of uncharged hydrophobic structures, they are unable to diffuse freely after they are released into the intracellular space. There are three possible ways AEA and 2-AG can diffuse into cells. One way is through transport proteins that can bind and translocate the endocannabinoids from one side of the cell. Another way is that the endocannabinoids can be taken up via simple diffusion driven by a concentration gradient established by intracellular enzymatic degradation. Lastly, the endocannabinoids can undergo endocytosis involving caveolae/lipid rafts [154]. AEA is degraded into free arachidonic acid and ethanolamine via the enzyme fatty acid amide hydrolase, while 2-AG is degraded into arachidonic acid and glycerol via the enzyme monoacylglycerol lipase [185].

The overall role of the cannabinoid system is somewhat complex. The endogenous cannabinoid system helps maintain normal body CNS functions such as memory, emotional processing, sleep, temperature homeostasis, pain, inflammation, hunger, and immunological responses while also contributing to pathological states such as anxiety, depression, schizophrenia, multiple sclerosis, neurodegeneration, and addiction [154]. The endogenous cannabinoid system also helps maintain PNS and peripheral tissue functions involving pain, energy, metabolism, and cardiovascular and reproductive functions while also contributing to pathological states of glaucoma, cancer, liver, and musculoskeletal disorders [154].

## 10. Physiological Effect of Cannabinoids

### 10.1. Cardiovascular Effects in the Context of Cannabinoid Use

Cannabinoids have several direct and indirect effects on cardiovascular system performance. The indirect effects are mediated by exposure to smoke and carboxyhemoglobin accumulation. Direct effects are primarily via direct CB receptors interaction and secondarily via sympathetic system activation.

Cannabinoids generally have vasodilatory reflex properties if they act through the CB1 receptor [186,187]. The response is complex and may consist of three phases with vagal-mediated hypotension (Phase I), followed by a compensatory increase in blood pressure (Phase II) to culminate in the prolonged hypotensive effect (Phase III) [188]. This latter effect is endothelially mediated and relies on nitrous oxide (NO), as vascular muscles respond with vasoconstriction to cannabinoids through unopposed CB2 interaction [109,186,189,190]. TRPV-1 is associated with the reflex bradycardia seen in Phase I, which has a cross function with CB1 receptors in Phase III to produce a hypotensive effect. PPAR and 5-HT_1a_ were also demonstrated as potential mediators for vasodilation [142,188,191,192,193]. The mechanism has likely central components [188]. What complicates studying this phenomenon is that anandamide results in triphasic effects, but other cannabinoids may not share all components. Being obese and using acetaminophen interfered with the vasorelaxant action of cannabinoids [186]. On the other hand, diabetes enhanced the expression and phosphorylation of CB1 receptors in the aorta with unclear clinical significance [194]. Hypertension resulted in varying degrees of cannabinoid impairments on vasorelaxation in the animal model [195,196]. The relaxation lasted for a minimum of 90 min, even in the secondhand intake [197]. Cannabinoids induce tachycardia by increasing the heart rate by approximately 20 to 60% as compared to baseline. The tachycardic response was linear to the dose, as dosages of 0.022 mg/kg THC and 0.044 mg/kg THC resulted in 36% and 69% elevation of heart rate from baseline, respectively. In a few cases, tachycardia was accompanied by orthostatic hypotension secondary to a decrease in the predominantly systolic component of blood pressure, but it was not a universal observation for all compounds [198,199]. These hypotensive effects occurred only at higher doses of cannabinoid antagonists [198]. The effect of illicit sympathomimetic additives such as cocaine has to be considered in patients with tachycardia after cannabinoid intake [200]. There is some habituation of tachycardia over weeks of using marijuana, but it was not statistically significant [201]. Ventricular performance was not affected by acute and prolonged cannabinoids in dogs even after 35 days of intake [202]. However, there are reports of cannabinoids causing cardiomyopathy [203].

Concomitantly, smoking one marijuana cigarette increases heart rate by 54% and reduces the time to the emergence of angina by 50% [129,171]. In another study, cardiovascular risk increased two-fold in young individuals consuming cannabinoids if their consumption was more than four times per month [204]. A similar excessive risk was seen in the case of a cardiovascular accident [205]. However, this is not a uniform finding [206]. It seems well established that an increased risk of a cardiovascular event in the wake of cannabinoid use is present even after singular exposure [207,208,209,210]. The increase in tachycardia, which may be a culprit in excessive risk of cardiac events, maybe potentially abolished by pretreatment with clonidine or propranolol, both central sympathomimetics [211,212]. However, since cannabinoids may induce tachycardia, resulting in oxygen delivery vs. supply mismatch, the coronary blood supply may be triggered by hypercoagulable states secondary to platelet activation. Furthermore, carboxyhemoglobinemia may be another contributing factor [82,170]. Finally, some suggested that the cardiotoxic effect of marijuana may be independent of classical culprits of the acute coronary syndrome [206].

### 10.2. Respiratory Effects in the Context of Cannabinoid Use

Cannabinoids may have a bronchodilator effect via interaction through the CB1 receptor. This interaction is strong enough for some to call for a new drug class designed for b-mimetic resistant individuals [122]. It is also possible that the anti-inflammatory effects of the CB2-mediate effect can facilitate the resolution of acute attacks [213].

THC was shown to significantly improve respiratory function in a study involving asthmatic human patients and healthy individuals [214,215,216]. The bronchodilator effect lasted for 60 min in healthy subjects if the cannabinoids were taken orally [215,216]. Furthermore, in the study involving asthma patients, 200 μg of THC was administered by aerosol to volunteer asthmatic patients at a stable state. Forced expiratory volume in one second (FEV) was recorded in 15-min intervals over the course of an hour after treatment. FEV at 15 min was approximately 0.2 L and FEV at 60 min was approximately 0.4 L [214]. This result shows a significant increase in *p* < 0.01 for THC. This study shows that a concentration of 200 μg of 9-THC in a 63-μg aerosol dose can be safely administered to asthmatic patients to improve respiratory function [214]. However, the benefits of this study proved to be very difficult to replicate, even though small studies confirmed the bronchodilator effect of cannabinoids in very small samples [98,217]. No tolerance to this effect over 20 days was observed [218]. Only 9-THC or 8-THC were pharmacologically active, while CBD and CBN were not [218]. These promising results are juxtaposed with the results produced by other clinical trials on the effect cannabinoids have on resolving bronchoconstriction. In COPD individuals, cannabinoids did not improve exercise tolerance, as demonstrated by a well-designed clinical trial [219]. Others demonstrated the irritable effect of 9-THC, outweighing an increase in large airway obstruction and the direct bronchodilator effect [219]. In another well-conducted study, males below 40 years old demonstrated significant decreases in expiratory flow rates at low lung volumes as well as the ratios of forced expiratory volume in one second to vital capacity; these decreases were to a larger degree than cigarette smokers [220]. In a Norwegian study, users of marijuana reported more use of bronchodilators if they had pre-existing lung conditions [70]. In addition, irritation, mucus production, and local inflammation are seen in cannabinoid smokers compared to healthy individuals and even tobacco users [221]. Hashish users seem to suffer from increased incidence of asthma and bronchoirritation. Consequently, smoking or vaping cannabinoid plants may lead to lung injury similar to that of smoking tobacco [173,222]. Marijuana smoking resulted in significantly higher carboxyhemoglobin and tar levels than regular smoking [82,170]. Exposure to cannabinoid smoke resulted in infiltration of neutrophils in the lung tissue [223]. In a longitudinal study of over 2000 young cannabinoid users, the risk of filling prescriptions was 1.72 times higher if an individual was a cannabinoid user [70]. Authors speculated this effect to be secondary to smoke-related irritation and inflammation. It is also likely that pyrolysis of cannabinoids produces bronchoconstrictor or carcinogenic substances. No conclusive data have demonstrated that cannabinoid smoking has the same health effects as tobacco cigarette smoking does, including COPD and cancer [224]. Vaping poses unique risks, as contaminants and additives to the vape medium may cause significant lung damage apart from cannabinoids [62,225]. These changes emerged even though cannabinoids are immune-inhibitory.

Respiratory drive was not affected by regular use of marijuana, but this was not uniform across different studies [128,201,226]. For example, in another study, is the consumption of two 900-mg cigarettes containing 2.2% marijuana resulted in a change of respiratory drive from baseline lasting 8 to 9 weeks [201]. These results are juxtaposed with the suggestion of the beneficial effect of cannabinoids in obstructive sleep apnea [106].

Finally, the case of cannabinoid allergy with compromised airways was described as well [227].

### 10.3. Neurological Effects in the Context of Cannabinoid Use

Cannabinoids have an ambivalent effect on cerebral blood flow. Under normal conditions, the CB1 receptor causes vasodilation with a subsequent increase in cerebral blood flow [228,229]. However, during hypoxia or hypercapnia, this effect changes to vasoconstricting. This effect may be responsible for the increased incidence of ischemic stroke among cannabinoid users [205,230]. In particular, young users have a 4.7-times-increased risk of stroke as compared to tobacco smokers [205]. Nevertheless, most of the strokes were survivable with good outcomes [231,232,233]. This is at least partially attributed to patients’ young ages. In addition, long-term exposure to cannabinoids results in morphological changes affecting the interpretation of blood flow [234]. This is one of the reasons why there is a complex interaction between the direct vasodilatory actions and the excessive risk of stroke in cannabinoid users [235]. The risk of stroke may increase even after singular exposure, with the risk lasting at least a week [210].

Furthermore, accelerated atherosclerosis and a high rate of intracranial artery stenosis were reported in cannabinoid users and could alternate causation of increased risk of strokes [231]. These data have been juxtaposed with experimental data, suggesting a neuroprotective mechanism of cannabinoid compounds after ischemia [236]. At least part of this neuroprotection is modulated by activation of peripheral MO via CB2 during ischemia [237]. In addition, modulation of neuroinflammation is frequently suggested as the potential beneficial mechanism of the cannabinoid to alter the natural history of neurodegenerative diseases [238,239,240]. On the other hand, prolonged heavy use of cannabinoids results in hippocampal thinning, neuronal death, and various complications [241,242,243,244].

Cannabinoids seem to have a protective effect on neuroinflammation. Downregulation of WNT, an inflammatory pathway, by cannabinoids was linked to improvement in inflammation and a decreased likelihood of Parkinson’s disease [13,245]. Subsequently, cannabinoids were studied and suggested as therapeutic for several neurodegenerative and neuroinflammatory symptoms [115]. While the use of cannabinoids appears to be therapeutic for several neurodegenerative and neuroinflammatory symptoms, no definitive evidence to date has been shown promoting their efficacy.

Pupils may be constricted during acute cannabinoid intoxication, but the effect is often compounded by impurities/adulterants of the cannabinoid’s preparation [246].

#### 10.3.1. Pain Perception in the Context of Cannabinoid Use

The effect of cannabinoids on pain perception is complex, and currently cannabinoids are not officially approved for pain treatment, although their use is permitted in several states per regional regulatory bodies in the USA.

Administration of 8-THC or CBD produced a significant reduction in pain scores in animals in response to capsaicin but only at high concentrations of both compounds: 0.5% and 1% for 8-THC and 5% for CBD. The mechanism of action for the different cannabinoids was then explored through various antagonists. Administration of the CB1R antagonist, AM251, blocked both the pain-mediating and the neutrophil-migrating effects of 8-THC, suggesting that 8-THC requires interaction with CB1R for its antinociceptive and anti-inflammatory effects. However, the same administration of AM251 did not affect the antinociceptive and anti-inflammatory effects of 5% CBD. Further research using CB2R−/− mice showed that CB2R is not involved in the antinociceptive or anti-inflammatory effects of THC or CBD [247]. Consequently, the CB1 receptor is implicated in direct pain mediation [118]. A noncannabinoid receptor, 5-HT_1A_, was shown to interact with cannabinoids and produce anti-inflammatory and antinociceptive effects. This interaction was confirmed by using a 5-HT_1A_ receptor antagonist, WAY100635, which blocked both the anti-inflammatory and antinociceptive effects. Additionally, modulation of the TPRV receptor by cannabinoids seems to be implicated in some pain modulation [120,134,135,137,141]. Finally, the anti-inflammatory effects of cannabinoid compounds also contribute to pain modulation [248,249,250].

Similar ambiguity is seen in human studies. The consensus is that cannabinoid compounds have direct and indirect nociceptive activity [64,79,248,250]. This pain modulation is particularly effective in chronic pain syndromes associated with neuropathic pain and inflammation [113,163,249,250,251,252]. The distinctive mechanism of action from opioids, nonsteroidal inflammatory medication, or acetaminophen makes cannabinoids an attractive adjunct even though the safety profile is not well established [79,112,253]. Finally, cannabinoids seem to reverse morphine tolerance in some cases [254]. Subsequently, nabilone was introduced to the market to treat pain as an adjunct medication [96,97,99,100]. However, these data need to be juxtaposed with the recreational use of cannabinoids. Marijuana had hyperalgesic activity and probably enhanced the perception of pain. In contrast, heavy smoking had little effect on discriminability and caused an increase in the pain report criterion (190). These effects persisted up to 4 weeks. Heavy consumption was 2% THC in 20 mg per 3–12 cigarettes per day (190). Differences in hosts’ expression of CB1 and CB2 receptors may be at fault in conjunction with the composition of marijuana smoke compounds.

The data demonstrated the efficiency of the cannabinoids in low back pain, headache, cancer, and surgical pain [112,250,255]. Cannabinoids may be a viable strategy to limit opioids since their effects are independent and addictive, considering their independent mechanism of action and less-addictive potential [251,256,257].

#### 10.3.2. Seizure Threshold in the Context of Cannabinoid Use

A decreased expression of CB2 receptors results in a lower seizure threshold [258,259,260]. Animal models and humans showed a beneficial effect of cannabinoids, particularly CBD, on seizure treatment. For example, 0.01 to 100 μM of CBD suppressed epileptiform activity in slices of the hippocampus region, as determined by the frequency of epileptiform local field potential (LFP) burst amplitude as well as burst duration [261]. One study applied this finding in human trials of patients with Lennox–Gastaut syndrome and Dravet syndrome, which are characterized by frequent seizures. Patients were tested with 20 mg/kg daily of cannabidiol. Of the 121 patients administered treatment, 43.9% reported a reduction of monthly drop seizure frequency. The cannabidiol was administered as an add-on therapy for treatment-resistant patients. Most studies testing the efficacy of cannabidiol as an anticonvulsive showed that less than 50% of patients proved to have reduced frequency of seizures, although most patients still experienced an adverse event. In this study, approximately 44% of the cannabidiol group experienced reduced seizures, whereas 86% of the same test group experienced adverse effects such as diarrhea, somnolence, pyrexia, decreased appetite, and vomiting. Furthermore, the extensive and complex metabolism of cannabinoids remains a problem with several interactions with other medications and significant effects of first-pass metabolism [90,174,262].

The benefits of cannabinoids have also been tested in seizure-like disorders. Initial studies focused on treating resistant seizures in the child population [263]. However, increasing data suggest that cannabinoids could be useful as treatment adjuncts [264,265,266,267]. The proposed mechanism of action is through CB2 receptors, resulting in hyperpolarization of surrounding membranes and the diminished effect of glycine toxicity [147,148,259,268]. Despite the enthusiasm and unwavering interest in using cannabinoids in epileptic treatment, the trials are limited to children and seizures resulting from neurodevelopmental, inherited and inflammatory disorders of the brain that are refractory to other treatments [266,268,269,270,271]. This somewhat cautionary approach may balance the hope that cannabinoids may provide multimodal mechanisms addressing several mechanisms underlying seizures (anti-inflammatory or metabolic) and the overall concern of side effects from these compounds [272].

### 10.4. Psychological, Behavioral, and Psychiatric Effects of Marijuana

There is significant euphoria/positive reinforcement during the intake of psychoactive cannabinoids combined with an anxiolytic effect [3,119,153,273,274]. The mechanisms involved with the activation of CB1 receptors by ligands (most commonly 9-THC) result in the reward’s reinforcement [119,275]. Activation of CB2 receptors seems to have the opposite effect [275]. Consequently, the effect of the cannabinoid compound will depend on the composition of its preparation (potency, enantiomer composition, and pharmacokinetics). Several regions of the brain are stimulated, particularly the mesolimbic dopaminergic system, followed by other parts of the brain, such as the posterior ventral tegmental area, the shell of the nucleus accumbens, and many others [273,274].

Cannabinoids influence the ability to make decisions in several settings. A synergistic relationship with alcohol showed impaired driving and nondriving skills [276]. Interestingly, willingness to be engaged in impaired activities was not changed [276]. This may suggest that cognitive impairment is very insidious.

What is of particular concern is the increased risk of psychiatric disorders. A longitudinal study of Swedish conscripts with more than 50 incidents of cannabinoid use resulted in a six-times-increased risk of emergence of schizophrenia in an independent way from other psychiatric disorders [277]. These psychiatric findings are similar to a previous study conducted in 1969 and other following studies. However, the emergence of psychologic changes may be time-delayed, as acute doses of cannabidiol ranging from 10 to 600 mg and chronic administration of 10 mg for 20 days or 3 mg/kg/day for 30 days did not induce psychologic or physical symptoms suggestive of psychotropic or toxic effects [278]. Cannabis influence on mood is very complex, further clouded by impurities of the cannabinoid’s preparations. Often the opposite effect is reported, including calming violent behavior induction [279]. However, this interaction is further complicated by prolonging several illicit substances and alcohol metabolism when taken with THC by interaction with microsomal hepatic system [280].

#### Overdose, Addictive Potential, and Withdrawal

Acute intoxication associated with cannabinoids is relatively uncommon but is of increasing frequency due to the introduction of synthetic cannabinoids into current preparations as well as the increase in 9-THC concentrations over time [281]. The effects are often compounded by adulterants added to the street preparation of cannabinoids. The effect of acute intoxication and the related “high” can be antagonized by physostigmine, as demonstrated in a few individuals [282]. Anxiety, hostility, paranoia, flushed eyes, erratic behavior, and tachycardia are commonly found [78]. However, these effects are unlikely to occur in cases of cannabidiol consumption [78,281].

THC has psychoactive components that contribute to its addictive potential [119,283]. 9-THC activates the mesolimbic system when ingested, causing an increase in free dopamine, which drives reinforcing and rewarding effects of the drug [119]. When rats were introduced to self-administration of THC directly into the posterior ventral tegmental area or the nucleus accumbent, the rats learned to lever-press to increase injections into the areas [274]. When THC was replaced with SR141716A, a CB1 receptor antagonist, the lever pressing responses were stopped [274]. This experiment shows the reinforcing properties of THC as well as the role the CB1 receptor plays in establishing such behaviors. With chronic administration of THC over time, tolerance develops by decreased densities of available CB1 receptors.

Dependence is defined as undesirable physical symptoms if a substance is suddenly stopped or taken in smaller quantities [283]. Common symptoms of THC withdrawal include anger, anxiety, decreased appetite, weight loss, irritability, restlessness, hostility, and sleep disturbance [283,284,285]. These symptoms usually occur 24 h after the last use, peak in 1–7 days, and last about 2–3 weeks [286,287]. It is estimated that 47% of regular users will experience withdrawal during their life [288]. Experiencing withdrawal symptoms is not considered life-threatening. Nabilone can be used in case-by-case cases to alleviate withdrawal symptoms, although this is an off-label use. In addition, dexmedetomidine was successfully used in one case report, suggesting that some of the symptoms of cannabinoid withdrawal may be secondary to cannabinoid interaction with the α2 receptor [289]. However, dexmedetomidine is used with several other withdrawal symptoms to lessen the severity of symptoms.

### 10.5. Coagulation and Cannabinoids

Endocannabinoids, specifically 2-AG, interact with other proinflammatory markers to influence hemostasis via cannabinoid and noncannabinoid pathways [290,291,292]. Platelet-activating factor (PAF), a potent mediator of inflammation, is released from activated neutrophils, macrophages, platelets, and endothelial cells. As local levels of PAF increase, studies show that macrophages, platelets, and endothelial cells release significant levels of endocannabinoid 2-AG but not anandamide [293]. When local levels of 2-AG increase, 2-AG is shown to interact with platelets through receptors outside of the common CB1/CB2 receptors. In the presence of a 2-AG CB-receptor agonist or antagonist, both fail to induce or prevent platelet aggregation, respectively, which was confirmed by the lack of CB1 or CB2 receptor mRNA in human platelets [126]. 2-AG promotes platelet shape change, granulation secretion, and increases in cytosolic Ca^2+^ and TxA2 formation, ultimately resulting in platelet aggregation [126]. Synthetic cannabinoids such as CBD do not have the same platelet activation and aggregation effect compared to the endocannabinoid anandamide [294]. Although anandamide can cause platelet activation, it does not activate platelets to the same magnitude as 2-AG. In the presence of PAF, anandamide is not generated by macrophages or platelets, thus supporting 2-AG being the primary endocannabinoid for hemostasis [293].

### 10.6. Liver Effects in the Context of Cannabinoid Use

Cannabinoids interact with the liver in several different ways. The role of liver metabolism of cannabinoids has been described above.

Several interactions with anesthetics are possible (Table 3). Cannabinoids will compete for CYP3A4 and CYP2C9 enzymes, resulting in a possible decrease in the metabolism of other medications utilizing the same enzymes. Diminished metabolism of ethanol and barbiturates was reported [295,296,297]. Polymorphisms in CYP2D6 may be of particular importance for competing for metabolism since cannabidiol significantly impairs this pathway [295]. Cytochrome P25C9 or 3A may have inhibitory influences on processing the cannabinoids and other anesthetics [296,297]. Secondly, cannabinoids significantly influence the immune system; thus, their presence may modulate several autoimmune and toxic processes by modulating the NF-κB pathway. Thirdly, paracetamol and cannabinoids have several intense interactions with difficult-to-judge clinical importance. Finally, there are suggestions of cannabinoids contributing to nonalcoholic liver disease, fibrosis, and inflammation, but the data are conflicting and inconclusive [298,299].

### 10.7. Immunology Performance in the Context of Cannabinoid Use

Cannabinoids have both direct and indirect effects on the immune system. The direct effects are mediated via CB2 cannabinoid receptors. The indirect effects are mediated by cannabinoids interacting with peripheral organs affecting immunological performance with CB1 and CB2 receptors variably involved. Other receptors may be involved as well [319]. Neuroimmunological connections are probably the most important in modulating immunologic responses, yet these connections are extremely difficult and complex.

Serum profiles of male cannabinoid users demonstrated a profound effect on IL-12-related activation patterns as well as the activation of MAPK kinases and NF-κB pathways [320]. These effects were complex, with diverse outcomes occurring on activation and inhibitory markers represented by 55 proteins being upregulated and 66 being downregulated. Of significance, liver X/retinoid X receptor (RXR) activation and acute phase response signaling were most affected when monitoring in vitro experiments [320,321]. In addition, activation of inflammasome 3 was significantly reduced by CBN [322]. This immunomodulatory profile suggests that cannabinoids may have a predominantly inhibitory effect on the immune system, which is reinforced by the reduction in IL-1β production [323].

Both CBD and THC affect lymphocyte proliferation, with CB1 and CB2 receptors playing important roles in immunomodulation [125,324]. THC primarily utilizes CB1 receptors, but it has been shown that CB2 receptors have the greatest effect in decreasing lymphocyte proliferation. Cannabinoid-induced lymphopenia causes the greatest decrease in T-cells, T-helper cells, cytotoxic T-cells, and B-cells in the lymphocyte subset. The ratio of Th1 to Th2 cells may be affected [325]. However, the total numbers of NK and NKT cells were independent to that of lymphocytes [326]. This concludes that cannabinoid-induced lymphopenia is somewhat a selective matter. CBD mostly inhibits T-cell invasion to an injury site. T-cell differentiation and invasion are closely linked to cytokine and chemokine levels. These cytokine levels are proinflammatory following an injury. When rats subjected to a spinal cord injury were given CBD, there was shown to be a decrease in cytokine levels and T-cell invasion. A similar suppressive effect is seen in monocytes and their response to bacterial and infection stimuli [238,324,327,328]. This immunosuppressive effect also extends to dendritic cells [329]. In a very elegant in vitro experiment, 9-THC was demonstrated to interrupt the interaction between T-cells and astrocytes, suggesting a potential dendritic cell-related immunosuppression [330].

These immune-modulatory properties of marijuana smoke have to be isolated from the effect of the smoke itself [170,219,223,224,331]. Cannabinoid compounds can also induce cell death at high concentrations [332].

The relevance of the immunosuppressive effect of cannabinoids is difficult to judge clinically. However, it may be responsible for increased susceptibility to pulmonary infection in recreational users and patients taking cannabinoid compounds for clinical conditions [90,91,92,325,333]. At the same time, antinociceptive and neuroprotective mechanisms are dependent on the anti-inflammatory action of cannabinoids [74,123,239,334,335,336].

## 11. Anesthetic Consideration for Cannabinoid Use

Most cannabinoid users are young. Consequently, their number of comorbidities is low, presenting a little challenge. However, on the other hand, a significant subgroup of individuals with significant cannabinoid intake are secondary to several medical, mostly chronic, severe conditions.

### Preoperative Period

**Figure 2:**
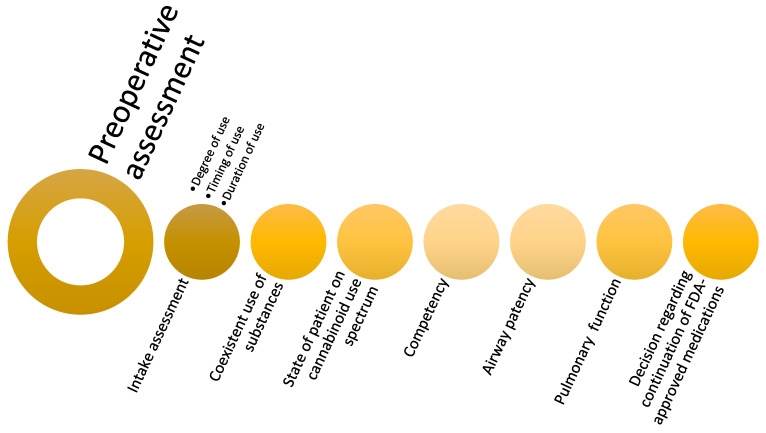
Preoperative consideration in anesthesia in an individual with cannabinoid intake.

Four main considerations present significant challenges for anesthesiologists during the perioperative period: (1) gauging the intake of cannabinoids, (2) evaluating the effects of cannabinoids, (3) assessing the competency to consent, and (4) management of cannabinoid medications (Figure 2).

A formidable challenge is gauging the intake of cannabinoids, as the dosages and potency of different cannabinoid preparations vary in strength and composition [18,19,35,37]. The standardization of the dosages is scant and complicated, with the concentration of 9-THC possibly being affected by 8-THC while being inversely related to CBD [77,78,83,179]. Furthermore, synthetic cannabinoids may further affect potency [10,207]. Considering the higher prevalence of other illicit drugs among cannabinoids, one must consider the possibility of multi-drug abuse with obvious implications for anesthesia management [9,200,276]. Cannabinoids have several pleiotropic properties [2,14,87,107,117,156,167,169,176]. Their intake varies greatly depending on the route of consumption, the consumers’ habits, the reason for consumption, and the corresponding effects on the cardiac, immunological, and nervous systems [17,26,44,46,71,72,256]. The lack of standardized tools available to providers to assess cannabinoid use compounds the problem. The high variability in cannabinoid preparation and biological actions effectively limits accurately gauging the risk related to cannabinoid intake during anesthesia management. With some of the effects of cannabinoids being nonlinear, it only adds to the difficulty with anesthesia management.

An individual may present in five potential states related to cannabinoid use: acute intoxication, chronic/habitual intake, withdrawal from cannabinoids substances, positive urine toxicology screen, or taking FDA-approved medication. The sign of acute intoxication includes symptoms of conjunctivitis, bloodshot eyes, dry mouth, tachycardia, and hypotension [337,338,339]. The demeanor is euphoric along with impairments to motor skills and short-term memory [67]. The patient may also present with excessive anxiety and paranoid ideation [67,337,338]. These symptoms may be difficult to recognize in individuals with second-hand exposure to cannabinoid compounds who cannot communicate effectively [337]. There is also a possibility that an intoxicated patient may suffer from a stroke, acutely masquerading intoxication [230]. The sign of chronic intake may be difficult to identify, considering that an individual may be partially habituated to cannabinoids’ effects while suffering from consequences of prolonged cannabinoid use. The classical urine test remains positive for days after cannabinoid intake, even if this was second-hand smoke or bystander intake. Only simultaneous blood draws to urine collection allow for assessing acute vs. chronic/historic exposure, as THC can last in the blood for 2 to 6 h [41,340,341,342]. In some instances, patients may present with signs of acute cannabinoid withdrawal centered around tachycardia and psychological disturbances (anxiety, hostility, etc.) [284,285]. The withdrawal manifests mostly with extreme agitation that may be managed conservatively with benzodiazepines, α2 agonists, and anxiolytics [285,286,289]. Long-term marijuana users can also suffer from hyperemesis syndrome [343]. Finally, the individual may be prescribed a cannabinoid-based medication in which most of them should continue through surgery [21,49,89,100].

When treating a patient suspected of cannabinoid use, it is very important to determine their ability to give consent properly. Cognitive impairment may be prolonged, making it difficult to judge the patient’s ability to give consent [276]. In general, intake of cannabinoids is related to impaired judgment but not risk avoidance [276,344]. Chronic exposure to cannabinoids results in morphological changes to the brain with an unclear ability to make decisions [234,345]. This is further complicated by the inability of current testing to recognize acute vs. delayed/chronic cannabinoid intake, as the metabolites measured in urine have a long half-life [180]. Finally, exposure to cannabinoids may occur in an insidious/second-hand way, impacting the ability to consent [66,68,230,346]. On the practical level, assessing an individual’s ability to render an informed decision should be undertaken and documented in the chart following standard practices.

In terms of cannabinoids affecting the assessment of perioperative fitness for surgery, cannabinoids have a complicated influence, and no studies exist showing if cannabinoid users suffer from an increased burden of ailments and illnesses. Overall, the effect of cannabinoids on the cardiovascular system is hypotension combined with tachycardia. Interestingly, cannabinoid concentrations increase in coronary arteries post-acute coronary syndrome, suggesting a beneficial or compensatory effect. This effect may be balanced against the risk of myocardial ischemia, platelet activation, cardiac arrhythmia, and cardiomyopathy [204,208,209,347]. Of note, increased risk of a coronary event is prolonged and occurs after even a single intake of cannabinoid compounds [204]. Concern regarding the respiratory system should focus on the potential effect of smoke and vape inhalation as an inducer of lung damage or affecting mucus production [62,73,172,173,225]. In addition, the upper airway should be evaluated for potential problems during intubation. In an interesting example, a case study regarding a 17-year-old male who smoked marijuana before his scheduled tympanomastoidectomy showed that his postoperative recovery was complicated by uvular edema, which led to blockage of his glottal opening corresponding to hypoxia [348]. In another case, necrosis of the uvula occurred during anesthesia in a cannabinoid user [349]. Other abnormalities of the oropharyngeal cavity may be present [350,351]. All possible causes for these complications to emerge, such as allergies and trauma, were considered, but eventually, cannabis use was decided to be the most likely cause [352].

Significant consideration should be devoted to the interaction of drugs with cannabinoids in current users. While some medications may have minimal interactions, cannabinoids interact extensively with protein binding and liver metabolism, affecting the level of other drugs and their metabolism.

## 12. Operative Period and Cannabinoids

**Figure 3:**
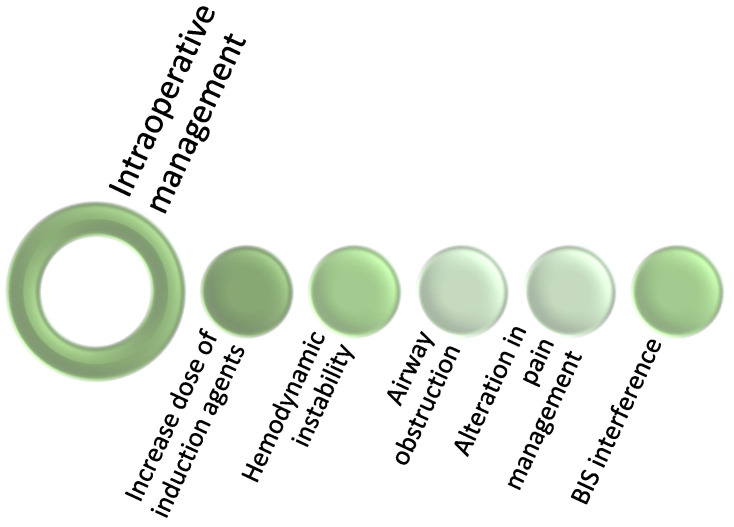
Intraoperative management of the individual with cannabinoid intake.

The effect of cannabinoid intake on intraoperative anesthesia management is mostly related to potential effect on sympathetic drive and airway management (Figure 3). Acute and chronic consumption of cannabinoids can have different effects on anesthetic requirements. In a human study examining the effects of reported cannabis use and propofol induction, patients reporting cannabis use for over a week required significantly higher doses of propofol to achieve a BIS <60% as well as higher doses of propofol to achieve endotracheal tube intubation [353]. The anesthesia induced by ketamine, thiopentone, or propanidid was prolonged if 9-THC was administered in mice [354,355]. This prolongation effect was not seen with the administration of cannabinol. However, pentobarbital was prolonged by cannabidiol [354]. One study suggested prolongation of ether anesthesia [355]. Another study demonstrated a reduction in MAC for sevoflurane, but only if intraperitoneal morphine at 5mg/kg was given concomitantly with cannabinoids to animals [356]. In rats, pretreatment with 9-THC reduced MAC sevoflurane by 26% [357]. However, another study in human subjects found higher BIS values in concurrent cannabinoid use [358]. Furthermore, in a double-blind, randomized control study, consumption of cannabinoids prior to surgery led to an increased average BIS during steady-state anesthesia^17^. Considering all the facts, cannabinoid consumption has increased EEG activity, rendering BIS a less reliable measurement of anesthetic depth while affecting the anesthesia requirements. No studies have explored the effects of cannabinoids on spinal, epidural, and regional anesthesia performance. Conversely, anesthesia may prolong the duration of cannabinoid metabolism, since it interferes with CYP3a metabolism [359].

With the increased prominence of laced marijuana, it is important to consider the effects of additive substances on cannabinoids. A case report of a patient with a history of smoking marijuana laced with amphetamines experienced an incidence of severe hypotension after intubation and administration of sevoflurane [360]. The patient’s hypotension was unresponsive to phenylephrine and a bolus of crystalloids. Eventually, the patient’s blood pressure was responsive and stabilized with the administration of epinephrine [360]. With increased consumption of cannabinoids worldwide, it is important to consider potential additives, as the patient may not be aware of them.

Acute cannabinoid use seems to be related to increased blood pressure, tachycardia, and increased or decreased cardiac contractility [128,129,196,198,199,200,202,206,207,232,361,362,363]. The limitation of these data is that the perioperative intake of cannabinoids is difficult to standardize even within the same patient. In addition, most of these effects are triggered by the interaction of the cannabinoids with the autonomic nervous system [128,207,363]. Consequently, it is unclear if a paradoxical hemodynamic response is possible in the case of a patient with sympathetic exhaustion while taking cannabinoids [127,129,361,362]. Concomitant intake of β-blockers may impact the cannabinoid-triggered responses. The type of response from prolonged consumption of cannabinoids is unclear, but some suggest an enhanced parasympathetic response [361]. The question of perioperative arrhythmias may be a more serious consideration, as arrhythmias were reported to be of higher incidence within cannabinoid users [364]. The risk of adverse cardiovascular events has not been evaluated definitively, but acute coronary syndrome and arrhythmias have been reported [208,348,364]. However, the risk of stroke seems to be the primary consideration in case series and metanalysis [232,363]. Acute coronary syndrome is limited to case reports and linked to increased platelet activation and autonomic instability [126,196,207,292]. Interestingly, endogenous cannabinoids are at an increased concentration in hypoperfusion syndrome and serve as coronary dilators [347]. The effect of cannabinoids on the pulmonary system seems to be limited to potential irritation, inflammation, and immune suppression [90,91,92,170,215,219,224]. Before intubation, a risk assessment should be conducted, focusing on potential swelling of the upper airway tissues and potential increased resistance of the lower airway [73,82,170,216,352]. During management, an increased risk of airway obstruction is present, especially in prolonged users of cannabinoids [170,215,216,219,224]. Should these complications emerge, they should be managed per standard of care (application of bronchodilators, suctioning, and bronchoscopy). No definite data exist linking cannabinoid use to clinically important effects on urinary, endocrine, and hepatic systems. It is unclear if cannabinoids are related to the emergence of malignant hyperthermia, but their intrinsic α2 activity suggests otherwise [94,130]. Thermoregulation seems to be intact, as one study demonstrates an identical incidence of shivering among marijuana users of a variety of levels [365]. The involvement of cannabinoids in lacrimation can place a patient at higher risk of corneal injury [366].

## 13. Postoperative Period and Cannabinoids

**Figure 4:**
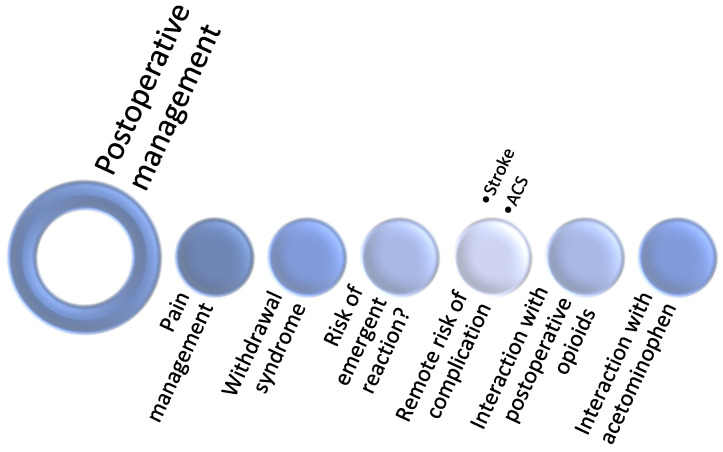
Postoperative consideration for cannabinoid users.

Postoperative management of patients who are cannabinoid users should be focused on: management of typical postoperative complications (nausea, vomiting, pain control, shivering, urinary incontinence and retention, and delirium), management of potential withdrawal symptoms, and mitigation of the risks associated with long-term cannabinoid use (Figure 4).

Despite the high potential of cannabinoids resulting in psychoactive disorders, there are no reports of increased frequency of emergence reaction after surgery, despite the increased risk of dementia, schizophrenia, and cognitive decline [277,345]. The effects of cannabinoids on postoperative pain management are not well established, despite some suggestions of using these compounds for chronic pain. Three studies demonstrated significant heterogeneity in effects [367,368,369]. These studies demonstrated that analgesic, antianalgesic, and hyperanalgesic effects of cannabinoids were partly related to their doses consumed. Though cannabinoids are not used as first-line treatment in pain management, experimental and clinical data show that concurrent cannabinoid use will have unpredictable effects on postoperative pain management with opioids. What could be of particular interest is the interaction of cannabinoids with opioids on respiratory depression, since there are at least some suggestions that cannabinoids can be utilized to improve obstructive sleep apnea [106,107,263]. Few cases are described, as there are potential liver injuries during concurrent cannabis and acetaminophen use [370]. THC and nabilone have both shown to have little to no effect on perioperative nausea and vomiting (PONV), even though the cannabinoids may directly mediate this effect [55,133,160]. A study involving THC to alleviate PONV symptoms revolved around the hypothesis that THC would reduce the risk of PONV by 25% compared to a placebo. Forty patients with high susceptibility to PONV were administered 0.125 mg/kg of THC or a placebo and were observed for 24 h after surgery. The primary outcome observed was the incidence of PONV. However, the study was discontinued due to the lack of efficiency of THC against PONV. In a study involving nabilone for PONV treatment, 340 participants were given either nabilone or a placebo, and the results showed that both subject groups experienced approximately a 20% likelihood of PONV. Both studies show that THC and nabilone have little effect on the symptoms of PONV when compared to a placebo. These are interesting findings, as some advocate utilizing cannabinoids to treat nausea in the wake of chemotherapy [48,55,97,160]. Location of the CB1 receptor on the prostate, vas deferens, and bladder suggest a potential role of cannabinoids in urinary retention, but clinical data are lacking except for a one-time remote report [371].

The emergence of postoperative complications in cannabinoid users is continuing to be established. In general, one study demonstrated a lack of major perioperative events in cannabinoid users. A total of 1818 patients consisting of cannabis users (606) and controls (1212) were analyzed and assessed in terms of perioperative complications [372]. No difference was found statistically significant between the two groups [372]. This study was completed through a retrospective electronic medical record review [372]. However, in a much larger study of over 27,000 records, the active cannabinoid user had increased odds of experiencing a myocardial infarction [373]. This finding is aligned with a prior observation of increased risk of cardiac events in cannabinoid users in general [129,196,206,232,361,362].

Finally, the potential for cannabinoid withdrawal should be assessed in conjunction with other potential withdrawal syndromes in polysubstance users. Cannabinoid withdrawal is usually unpleasant but rarely results in difficult complications.

## 14. Limitations

The above overview is a very basic guide due to the paucity of recent data and the complex nature of cannabinoids’ biological properties. The FDA’s classification of marijuana as an illicit substance resulted in a paucity of research. Consequently, several studies cited are 40 years old or older. Several studies are in vitro or in animals. Their translation in the effect of anesthesia performance during surgery may be limited. Most current evidence is related to case reports or case series with obvious limitations. The effect of the anesthetic cannot be discerned from other illicit substances because of a lack of a reliable way to distinguish the dose, composition, and timing of cannabis compounds. Of note, cannabinoid users, most of the time, are not privy to this information, forcing anesthesiologists to work in the dark.

## 15. Conclusions

Cannabinoids are biologically active compounds with highly unstandardized and complex chemistry. Lack of standardized tools and modern knowledge affects the ability of anesthesiologists to predict the effects of cannabinoids on anesthesiology execution accurately. The effects may be substantial but nonlinear, adding another layer of complexity. Though cannabinoid intake should be considered, anesthesia plans should be executed carefully and take into account the potential effects of cannabinoids. The FDA-approved cannabinoid compounds should be continued during treatment.

## Figures and Tables

**Table 1:** Naturally and artificially synthesized cannabinoids and their main biological effects. Additionally included are the specific cannabinoid receptor and type of receptor which are utilized.

Cannabinoid	Predominant Receptor(s)	Type(s) of Binding	Dominant Effects in Humans	Ref.
Δ9-THC	CB1, CB2	Partial agonist	Psychotropic effect, appetite, analgesia	[75]
Δ8-THC	CB1, CB2	Partial agonist	Psychotropic effect, appetite, analgesia	[58,75]
Cannabidiol (CBD)	CB1, CB2, α_2_, 5-HT_1A_	Agonist	anti-inflammatory, analgesic, antianxiety, antitumor	[49,75,89]
Cannabinol (CBN)	CB1, CB2	Partial agonist, antagonist	Immune suppression, seizure suppression	[49,75,89,90,91,92]
Cannabichromene (CBC)	TRPV1, CB2	Agonist	Antinociceptive, anti-inflammatory	[85]
Cannabicitran (CBT)	CB1?, CB2?	Unclear	Poorly defined	[75]
Cannabidivarin (CBDV)	CB1, CB2	Antagonist	Antipsychotic effect of metabolites	[75,93]
Cannabigerol (CBG)	CB1, α_2_, 5-HT_1A_	Antagonist, agonist	Antitumor activity, poorly defined	[94]
Nabilone	CB1	Agonist	Pain adjunct, appetite stimulation	[21,95,96,97,98,99,100,101]
Nabiximols	CB1, CB2	Partial Agonist	Alleviating symptoms of dry mucosa in multiple sclerosis	[47,102]
Dronabinol	CB1, CB2	Partial Agonist	Appetite stimulation	[103,104,105,106,107]
Rimonabant/SR141716	CB1	Antagonist	Appetite inhibition, seizure threshold, psychoactive	[50,108,109]

**Table 2:** Cannabinoid receptors, agonists, locations, and actions in humans.

Receptor	Ligands	Dominant Location	Dominant Action	Ref.
CB1	Anandamide, THC 2-AG, 2-AGE, SR141716	CNS	Pain, memory, energy metabolism	[88,108,116,117,118,119,120,121,122,123,124]
CB2	Anandamide, THC 2-AG, CBDV	CNS, immune cells	Immune signaling, inflammatory responses	[93,116,125,126]
Α2	Norepinephrine, CBG	CNS	Central sympathectomy, vasodilatory, antianxiolytic effects	[14,127,128,129,130,131]
5-HT_1A_	Serotonin, CBD	CNS, platelets	Mood, platelet activation, antinausea	[94,132,133]
TRPV	Anandamide, CBC, ODA, NADA	PNS	Pain, inflammation modulation, vascular tone	[120,134,135,136,137,138,139,140,141,142,143]
GLYR	Glycine, THC	CNS	Motor control, pain, synaptic neurotransmission, dependence, cholesterol membrane metabolism	[144,145,146,147,148]

**Table 3:** Interaction between cytochromes and anesthetic agents while being modulated by cannabinoids.

Cytochrome	Cannabinoid	Cannabinoid Effect	Anesthetic
CYP2B6	THC, CBD	THC and CBD both are inhibitors [300]	Propofol [301], Ketamine [302,303]
CYP2C9	THC, Cannabinol [304]	THC as inhibitor [300,305]	Propofol [301], Ketamine [302,303], Rocuronium [306]
CYP2E1	THC metabolites, CBD	Competetive inhibition [300]	Halothane [307], Isoflurane [307], Sevoflurane [307], Enflurane [307], Desflurane [307,308]
CYP3A4	THC, CBD, Cannabinol	CBD is an inhibitor [300,309]	Ketamine [302], Midazolam [310], Diazepam [311], Fentanyl [312], Rocuronium [306], Codeine [307,313], Propofol [301], Acetaminophen [314]
CYP2A6	THC	Genotype-dependent [295]	Dexmedetomidine [315]
CYP2C19	THC, CBD	THC and CBD are both inhibitors [309]	Diazepam [311], Rocuronium [306]
CYP1A1/2	CBD	CBD is an inducer [309]	Diazepam [311]
CYP2D6	THC, CBD	THC and CBD are both inhibitors [300]	Tramadol [316], Codeine [314,317], Oxycodone [314,318], Hydrocodone [303,318], Methadone [303,318]

## Data Availability

The datasets used and/or analyzed during the current study are available from the corresponding authors on reasonable request.

## Abbreviations

2-AG	2-arachidonoylglycerol
5-HT1A	5-hydroxytryptamine
8-THC	(−)-trans-Δ8-tetrahydrocannabinol
9-THC	(−)-trans-Δ9-tetrahydrocannabinol
AEA	arachidonoylethanolamide
cAMP	cyclic adenosine monophosphate
CB1	Cannabinoid receptor 1
CB2	Cannabinoid receptor 2
CBC	Cannabichromene
CBD	Cannabidiol
CBN	Cannabinol
CYP-450	cytochrome P450
DOR	δ-opioid receptor
GDP	guanosine diphosphate
GTP	guanosine triphosphate
MOR	μ-opioid receptor
NK	natural killer
THC	tetrahydrocannabinol
NADA	N-arachidonoyl-dopamine
ODA	N-oleoyl-dopamine
GLYR	Glycine Receptor
